# Supplementary material for: Endometriosis Associated-miRNome Analysis of Blood Samples: A Prospective Study
Source: Diagnostics (Basel). 2022 May 5;12(5):1150. doi: 10.3390/diagnostics12051150 (PMC9140062; doi:10.3390/diagnostics12051150)
Supplement: Supplementary file 1 [file diagnostics-12-01150-s001.zip › diagnostics-1652980-supplementary.pdf]

Table S1. Performances of the 229 differentially expressed in the cohort.

miRNAs	AUC	F1-Score	Sensitivity	Specificity	Up or Down
hsa-miR-224-5p	0.459	0.568	0.461	0.457	UP
hsa-miR-3135b	0.577	0.773	0.74	0.413	UP
hsa-miR-11400	0.567	0.76	0.721	0.413	UP
hsa-miR-100-3p	0.532	0.226	0.13	0.935	UP
hsa-miR-10397-5p	0.539	0.246	0.143	0.935	UP
hsa-miR-1180-5p	0.564	0.867	0.955	0.174	DOWN
hsa-miR-1183	0.533	0.197	0.11	0.957	UP
hsa-miR-1185-5p	0.544	0.827	0.87	0.217	DOWN
hsa-miR-12113	0.526	0.871	0.987	0.065	DOWN
hsa-miR-12115	0.549	0.869	0.968	0.13	DOWN
hsa-miR-1231	0.55	0.861	0.948	0.152	DOWN
hsa-miR-1253	0.578	0.847	0.896	0.261	DOWN
hsa-miR-1256	0.568	0.408	0.266	0.87	UP
hsa-miR-1267	0.528	0.856	0.948	0.109	DOWN
hsa-miR-1286	0.536	0.133	0.071	1	UP
hsa-miR-1298-3p	0.544	0.198	0.11	0.978	UP
hsa-miR-135a-2-3p	0.544	0.198	0.11	0.978	UP
hsa-miR-146a-3p	0.577	0.609	0.481	0.674	UP
hsa-miR-153-5p	0.546	0.779	0.766	0.326	DOWN
hsa-miR-181b-3p	0.549	0.178	0.097	1	UP
hsa-miR-1910-5p	0.555	0.875	0.981	0.13	DOWN
hsa-miR-1973	0.529	0.874	0.994	0.065	DOWN
hsa-miR-203a-5p	0.5	0	0	1	DOWN
hsa-miR-203b-3p	0.52	0.154	0.084	0.957	UP
hsa-miR-208a-3p	0.579	0.818	0.831	0.326	DOWN
hsa-miR-208a-5p	0.569	0.863	0.942	0.196	DOWN
hsa-miR-210-5p	0.582	0.418	0.273	0.891	UP
hsa-miR-211-3p	0.553	0.42	0.279	0.826	UP
hsa-miR-216b-3p	0.551	0.879	0.994	0.109	DOWN
hsa-miR-219a-5p	0.586	0.684	0.584	0.587	UP
hsa-miR-2681-3p	0.567	0.268	0.156	0.978	UP
hsa-miR-29b-1-5p	0.68	0.781	0.708	0.652	UP
hsa-miR-3059-3p	0.526	0.099	0.052	1	UP
hsa-miR-3064-3p	0.574	0.851	0.909	0.239	DOWN
hsa-miR-3085-5p	0.541	0.87	0.974	0.109	DOWN
hsa-miR-3122	0.59	0.333	0.201	0.978	UP
hsa-miR-3128	0.598	0.456	0.305	0.891	UP
hsa-miR-3129-5p	0.549	0.412	0.273	0.826	UP
hsa-miR-3137	0.617	0.779	0.734	0.5	DOWN

hsa-miR-3140-5p	0.546	0.237	0.136	0.957	UP
hsa-miR-3150a-5p	0.5	0	0	1	DOWN
hsa-miR-3155a	0.52	0.154	0.084	0.957	UP
hsa-miR-3156-5p	0.542	0.156	0.084	1	UP
hsa-miR-3159	0.563	0.82	0.844	0.283	DOWN
hsa-miR-3168	0.618	0.803	0.779	0.457	DOWN
hsa-miR-3180-5p	0.539	0.246	0.143	0.935	UP
hsa-miR-3185	0.566	0.878	0.981	0.152	DOWN
hsa-miR-3189-3p	0.532	0.122	0.065	1	UP
hsa-miR-3193	0.551	0.218	0.123	0.978	UP
hsa-miR-3197	0.568	0.844	0.896	0.239	DOWN
hsa-miR-323a-5p	0.55	0.247	0.143	0.957	UP
hsa-miR-33b-3p	0.543	0.227	0.13	0.957	UP
hsa-miR-346	0.415	0.725	0.721	0.109	UP
hsa-miR-3617-5p	0.57	0.502	0.357	0.783	UP
hsa-miR-3619-3p	0.593	0.342	0.208	0.978	UP
hsa-miR-3620-3p	0.563	0.286	0.169	0.957	UP
hsa-miR-3622a-3p	0.5	0	0	1	DOWN
hsa-miR-3648	0.574	0.823	0.844	0.304	DOWN
hsa-miR-3663-3p	0.529	0.874	0.994	0.065	DOWN
hsa-miR-3664-5p	0.556	0.267	0.156	0.957	UP
hsa-miR-370-5p	0.549	0.178	0.097	1	UP
hsa-miR-376a-5p	0.55	0.335	0.208	0.891	UP
hsa-miR-376b-5p	0.565	0.321	0.195	0.935	UP
hsa-miR-376c-5p	0.585	0.349	0.214	0.957	UP
hsa-miR-380-5p	0.528	0.144	0.078	0.978	UP
hsa-miR-3907	0.56	0.337	0.208	0.913	UP
hsa-miR-3923	0.557	0.868	0.961	0.152	DOWN
hsa-miR-3928-5p	0.547	0.858	0.942	0.152	DOWN
hsa-miR-3944-3p	0.54	0.851	0.929	0.152	DOWN
hsa-miR-410-5p	0.539	0.145	0.078	1	UP
hsa-miR-411-5p	0.558	0.699	0.617	0.5	UP
hsa-miR-4261	0.595	0.864	0.929	0.261	DOWN
hsa-miR-4290	0.541	0.187	0.104	0.978	UP
hsa-miR-4307	0.534	0.87	0.981	0.087	DOWN
hsa-miR-4421	0.536	0.236	0.136	0.935	UP
hsa-miR-4424	0.543	0.227	0.13	0.957	UP
hsa-miR-4430	0.574	0.424	0.279	0.87	UP
hsa-miR-4433a-3p	0.558	0.384	0.247	0.87	UP
hsa-miR-4436b-3p	0.566	0.45	0.305	0.826	UP
hsa-miR-4468	0.527	0.175	0.097	0.957	UP

hsa-miR-4474-3p	0.556	0.427	0.286	0.826	UP
hsa-miR-4477b	0.59	0.525	0.377	0.804	UP
hsa-miR-4484	0.552	0.44	0.299	0.804	UP
hsa-miR-4493	0.547	0.301	0.182	0.913	UP
hsa-miR-4502	0.57	0.437	0.292	0.848	UP
hsa-miR-4512	0.56	0.871	0.968	0.152	DOWN
hsa-miR-4518	0.548	0.208	0.117	0.978	UP
hsa-miR-4524a-5p	0.529	0.11	0.058	1	UP
hsa-miR-4524b-5p	0.565	0.321	0.195	0.935	UP
hsa-miR-4525	0.595	0.738	0.669	0.522	UP
hsa-miR-4531	0.55	0.247	0.143	0.957	UP
hsa-miR-4536-3p	0.549	0.178	0.097	1	UP
hsa-miR-4536-5p	0.555	0.293	0.175	0.935	UP
hsa-miR-4638-3p	0.538	0.357	0.227	0.848	UP
hsa-miR-4639-3p	0.552	0.188	0.104	1	UP
hsa-miR-4642	0.565	0.4	0.26	0.87	UP
hsa-miR-4645-5p	0.577	0.805	0.805	0.348	DOWN
hsa-miR-4647	0.539	0.145	0.078	1	UP
hsa-miR-4649-5p	0.532	0.122	0.065	1	UP
hsa-miR-4674	0.57	0.797	0.792	0.348	DOWN
hsa-miR-4679	0.533	0.197	0.11	0.957	UP
hsa-miR-4688	0.587	0.454	0.305	0.87	UP
hsa-miR-4689	0.577	0.452	0.305	0.848	UP
hsa-miR-4693-3p	0.5	0	0	1	DOWN
hsa-miR-4701-3p	0.544	0.198	0.11	0.978	UP
hsa-miR-4703-3p	0.539	0.145	0.078	1	UP
hsa-miR-4703-5p	0.551	0.879	0.994	0.109	DOWN
hsa-miR-4709-3p	0.532	0.122	0.065	1	UP
hsa-miR-4712-3p	0.557	0.328	0.201	0.913	UP
hsa-miR-4715-5p	0.587	0.324	0.195	0.978	UP
hsa-miR-4719	0.57	0.363	0.227	0.913	UP
hsa-miR-4724-5p	0.527	0.175	0.097	0.957	UP
hsa-miR-4725-5p	0.537	0.874	0.987	0.087	DOWN
hsa-miR-4726-5p	0.557	0.328	0.201	0.913	UP
hsa-miR-4740-5p	0.5	0	0	1	DOWN
hsa-miR-4747-3p	0.541	0.187	0.104	0.978	UP
hsa-miR-4748	0.605	0.714	0.623	0.587	UP
hsa-miR-4749-5p	0.58	0.777	0.747	0.413	DOWN
hsa-miR-4750-3p	0.529	0.874	0.994	0.065	DOWN
hsa-miR-4758-5p	0.575	0.814	0.825	0.326	DOWN
hsa-miR-4762-5p	0.572	0.84	0.883	0.261	DOWN

hsa-miR-4764-5p	0.529	0.874	0.994	0.065	DOWN
hsa-miR-4765	0.561	0.249	0.143	0.978	UP
hsa-miR-4779	0.545	0.265	0.156	0.935	UP
hsa-miR-4783-3p	0.539	0.246	0.143	0.935	UP
hsa-miR-4784	0.568	0.537	0.396	0.739	UP
hsa-miR-4787-3p	0.546	0.865	0.961	0.13	DOWN
hsa-miR-4796-5p	0.587	0.865	0.935	0.239	DOWN
hsa-miR-487a-3p	0.56	0.337	0.208	0.913	UP
hsa-miR-5001-5p	0.528	0.809	0.838	0.217	UP
hsa-miR-5004-3p	0.562	0.882	0.994	0.13	DOWN
hsa-miR-5007-5p	0.555	0.199	0.11	1	UP
hsa-miR-503-3p	0.541	0.438	0.299	0.783	UP
hsa-miR-504-3p	0.567	0.871	0.961	0.174	DOWN
hsa-miR-5089-3p	0.532	0.86	0.955	0.109	DOWN
hsa-miR-509-3-5p	0.529	0.11	0.058	1	UP
hsa-miR-513b-3p	0.543	0.227	0.13	0.957	UP
hsa-miR-514b-5p	0.555	0.875	0.981	0.13	DOWN
hsa-miR-515-3p	0.486	0.839	0.929	0.043	UP
hsa-miR-515-5p	0.604	0.422	0.273	0.935	UP
hsa-miR-517c-3p	0.454	0.072	0.039	0.87	DOWN
hsa-miR-5192	0.583	0.529	0.383	0.783	UP
hsa-miR-519d-3p	0.53	0.867	0.974	0.087	DOWN
hsa-miR-520d-3p	0.545	0.167	0.091	1	UP
hsa-miR-525-5p	0.516	0.063	0.032	1	UP
hsa-miR-541-3p	0.535	0.166	0.091	0.978	UP
hsa-miR-548as-3p	0.564	0.346	0.214	0.913	UP
hsa-miR-548as-5p	0.562	0.507	0.364	0.761	UP
hsa-miR-548av-5p	0.592	0.441	0.292	0.891	UP
hsa-miR-548j-3p	0.586	0.591	0.455	0.717	UP
hsa-miR-548j-5p	0.628	0.616	0.474	0.783	UP
hsa-miR-551b-3p	0.552	0.188	0.104	1	UP
hsa-miR-568	0.487	0.831	0.909	0.065	UP
hsa-miR-5688	0.539	0.145	0.078	1	UP
hsa-miR-5698	0.549	0.479	0.338	0.761	UP
hsa-miR-5703	0.557	0.868	0.961	0.152	DOWN
hsa-miR-573	0.54	0.851	0.929	0.152	DOWN
hsa-miR-597-5p	0.533	0.197	0.11	0.957	UP
hsa-miR-600	0.535	0.166	0.091	0.978	UP
hsa-miR-601	0.585	0.854	0.909	0.261	DOWN
hsa-miR-605-3p	0.537	0.299	0.182	0.891	UP
hsa-miR-6070	0.568	0.844	0.896	0.239	DOWN

hsa-miR-6074	0.548	0.208	0.117	0.978	UP
hsa-miR-6075	0.562	0.856	0.929	0.196	DOWN
hsa-miR-6081	0.588	0.857	0.916	0.261	DOWN
hsa-miR-6082	0.542	0.156	0.084	1	UP
hsa-miR-6084	0.534	0.844	0.916	0.152	DOWN
hsa-miR-6132	0.5	0	0	1	UP
hsa-miR-616-5p	0.582	0.6	0.468	0.696	UP
hsa-miR-649	0.524	0.834	0.896	0.152	DOWN
hsa-miR-6502-5p	0.603	0.443	0.292	0.913	UP
hsa-miR-6507-5p	0.537	0.299	0.182	0.891	UP
hsa-miR-6513-5p	0.571	0.544	0.403	0.739	UP
hsa-miR-659-3p	0.539	0.145	0.078	1	UP
hsa-miR-6722-3p	0.556	0.267	0.156	0.957	UP
hsa-miR-6726-5p	0.556	0.267	0.156	0.957	UP
hsa-miR-6731-3p	0.554	0.229	0.13	0.978	UP
hsa-miR-6733-5p	0.556	0.849	0.916	0.196	DOWN
hsa-miR-676-5p	0.52	0.154	0.084	0.957	UP
hsa-miR-6761-3p	0.542	0.156	0.084	1	UP
hsa-miR-6780b-3p	0.532	0.316	0.195	0.87	UP
hsa-miR-6786-5p	0.586	0.591	0.455	0.717	UP
hsa-miR-6787-3p	0.54	0.217	0.123	0.957	UP
hsa-miR-6788-3p	0.545	0.873	0.981	0.109	DOWN
hsa-miR-6788-5p	0.545	0.167	0.091	1	UP
hsa-miR-6790-5p	0.542	0.256	0.149	0.935	UP
hsa-miR-6794-5p	0.56	0.337	0.208	0.913	UP
hsa-miR-6795-5p	0.557	0.328	0.201	0.913	UP
hsa-miR-6797-5p	0.538	0.176	0.097	0.978	UP
hsa-miR-6799-3p	0.564	0.867	0.955	0.174	DOWN
hsa-miR-6805-3p	0.522	0.121	0.065	0.978	UP
hsa-miR-6806-5p	0.561	0.837	0.883	0.239	DOWN
hsa-miR-6809-3p	0.581	0.858	0.922	0.239	DOWN
hsa-miR-6810-5p	0.564	0.422	0.279	0.848	UP
hsa-miR-6811-3p	0.549	0.869	0.968	0.13	DOWN
hsa-miR-6817-5p	0.548	0.359	0.227	0.87	UP
hsa-miR-6819-3p	0.544	0.198	0.11	0.978	UP
hsa-miR-6821-3p	0.534	0.87	0.981	0.087	DOWN
hsa-miR-6822-3p	0.518	0.872	0.994	0.043	DOWN
hsa-miR-6824-3p	0.552	0.872	0.974	0.13	DOWN
hsa-miR-6825-3p	0.538	0.176	0.097	0.978	UP
hsa-miR-6840-3p	0.526	0.871	0.987	0.065	DOWN
hsa-miR-6847-3p	0.531	0.155	0.084	0.978	UP

hsa-miR-6855-3p	0.584	0.667	0.558	0.609	UP
hsa-miR-6860	0.531	0.254	0.149	0.913	UP
hsa-miR-6865-5p	0.565	0.321	0.195	0.935	UP
hsa-miR-6869-3p	0.516	0.174	0.097	0.935	UP
hsa-miR-6875-3p	0.553	0.865	0.955	0.152	DOWN
hsa-miR-6879-5p	0.579	0.479	0.331	0.826	UP
hsa-miR-6890-5p	0.58	0.306	0.182	0.978	UP
hsa-miR-7106-5p	0.539	0.145	0.078	1	UP
hsa-miR-7107-3p	0.532	0.833	0.89	0.174	DOWN
hsa-miR-7108-3p	0.594	0.816	0.818	0.37	DOWN
hsa-miR-7109-5p	0.549	0.869	0.968	0.13	DOWN
hsa-miR-7111-5p	0.538	0.273	0.162	0.913	UP
hsa-miR-7113-3p	0.554	0.229	0.13	0.978	UP
hsa-miR-7150	0.57	0.855	0.922	0.217	DOWN
hsa-miR-7152-5p	0.5	0	0	1	DOWN
hsa-miR-7156-5p	0.483	0.835	0.922	0.043	UP
hsa-miR-762	0.567	0.354	0.221	0.913	UP
hsa-miR-767-5p	0.513	0.051	0.026	1	UP
hsa-miR-7852-3p	0.544	0.198	0.11	0.978	UP
hsa-miR-8073	0.549	0.842	0.903	0.196	DOWN
hsa-miR-8086	0.546	0.237	0.136	0.957	UP
hsa-miR-887-5p	0.529	0.216	0.123	0.935	UP
hsa-miR-889-5p	0.563	0.875	0.974	0.152	DOWN
hsa-miR-943	0.534	0.264	0.156	0.913	UP
hsa-miR-9899	0.544	0.855	0.935	0.152	DOWN
hsa-miR-9901	0.526	0.206	0.117	0.935	UP
